# Breath, brain, and performance: investigating the effects of respiratory patterns on anxiety and cognition in bassoon players

**DOI:** 10.3389/fpsyt.2026.1786605

**Published:** 2026-07-15

**Authors:** Guang Yang, Heng Chen, Shuai Yang

**Affiliations:** 1 Zhejiang Conservatory of Music, Hangzhou, China; 2 University of Malaya, Kuala Lumpur, Malaysia; 3 Zhengzhou University Henan Conservatory of Music, Zhengzhou, China

**Keywords:** attentional control, bassoon, dysfunctional breathing, music performance anxiety, wind musicians

## Abstract

Music performance anxiety (MPA) represents a prevalent challenge among musicians, yet the specific relationships between respiratory patterns, anxiety, and cognitive function in wind instrumentalists remain underexplored. This cross-sectional study investigated these relationships in bassoon players, a population facing unique respiratory demands due to the instrument’s double reed mechanism and sustained breath support requirements. A total of 118 bassoon players (48 professional, 70 amateur) completed an online questionnaire comprising the Self-Evaluation of Breathing Questionnaire (SEBQ), Kenny Music Performance Anxiety Inventory-Revised (K-MPAI-R), Attentional Control Scale (ACS), and self-rated performance measures. Pearson correlation analysis revealed that dysfunctional breathing was positively associated with performance anxiety (r = 0.52, p < 0.001) and negatively associated with attentional control (r = -0.39, p < 0.001). Hierarchical regression indicated that dysfunctional breathing explained 23.1% of variance in performance anxiety beyond demographic covariates. Mediation analysis using bootstrapping demonstrated that performance anxiety partially mediated the relationship between dysfunctional breathing and attentional control, with the indirect effect accounting for 59.4% of the total effect (ab = -0.19, 95% CI [-0.30, -0.10]). Independent samples t-tests showed that professional players exhibited lower dysfunctional breathing scores (d = 0.58), higher attentional control (d = 0.54), and better performance outcomes compared to amateur players; however, performance anxiety levels did not differ significantly between groups (p = 0.448). These findings support the application of Attentional Control Theory to wind instrument performance contexts and highlight respiratory patterns as a potential intervention target for managing MPA. The results suggest that breathing-focused training may benefit bassoon players by reducing anxiety and enhancing cognitive function, though longitudinal research is needed to establish causal relationships.

## 1 Introduction

Music performance anxiety (MPA) is a widespread psychological problem that affects musicians of all levels. There have been epidemiological studies that have shown that it affects professionals between 16.5% and 60%, whereas students rate between 21% and 50% (1, 2). Debilitating cases of music performance anxiety (MPA) affect between 60% and 80% of professional musicians, with about 95% of performers experiencing stage anxiety during live performances (3). MPA occurs through its affective, cognitive, and somatic symptoms, which include excessive worry, fear of negative evaluation, distraction, and somatic symptoms of tremors, sweating, and breathing difficulties (4, 5). The impact of MPA can be significantly damaging to musicians in terms of performance, career, and mental health (6).

Wind instrument performers face their own set of physiological demands, which are different from those of other musicians, because the generation of sound through these instruments requires precise respiratory regulation and coordination of the breathing muscles and embouchure formation (7, 8). Playing a wind musical instrument is described as a very strenuous breathing function that demands control of air exhalation as well as creating levels of pressure characteristic of each musical instrument (9). With respect to the woodwind instruments, the bassoon is one of the most distinctive instruments in terms of breathing difficulties caused by the double reed and the production of notes in the lower register of the bassoon (10). In the playing of the bassoon, the techniques of abdominal breathing, thoracic breathing, and thoracic and abdominal breathing are highlighted as the activation of the diaphragm and the abdominal muscles to expand and contract the chest to create and release the airflow column (11). Somatic complaints relating to breathing are significantly more prevalent in wind instrument players compared to those playing strings, pianists, and percussionists, which suggests that breathing issues are an important source of concern for wind instrument players (12).

Slowing down the breathing process can have a significant impact in lessening the symptoms of anxiety by promoting the activities of the parasympathetic nervous system and by improving heart rate variability (13, 14). Breathing at the resonant frequency, which is six cycles per minute, can lower stress levels, improve mood, and boost cognitive functioning in young individuals (15). Resonance breathing techniques focused on the individual’s resonant frequency show an increase in the respiratory sinus arrhythmia, providing information on the relationship between sympathetic and parasympathetic activity of the nervous system (16). There is a bidirectional relationship between breathing and emotions, and anxiety affects breathing patterns, increasing breathing rate with increasing cognitive and mental loads (17). As for the wind instrumentalists, this relationship assumes the added importance because irregular breathing not only affects the psychological well-being, it also hampers the technical aspect of sound production while playing the instrument. Breathing exercises along with conventional therapy have proved to be effective in reducing the breathing irregularities and the level of anxiety in people with generalized anxiety disorder (18).

Attentional Control Theory provides a strong theoretical foundation for understanding the cognitive effects of anxiety within a performance context (19, 20). Anxiety interferes with the effective functioning of the goal-driven attentional system, while simultaneously increasing the stimulus-driven attention to threat-related stimuli. Two executive functions, namely inhibition and cognitive shifting, are also profoundly impacted, thus interfering with working memory and attentional flexibility (20). This theory also suggests that anxiety may not necessarily impact performance effectiveness (i.e., the quality of performance) when strategies such as increased effort are employed, but it always impairs processing efficiency (i.e., the relationship between performance effectiveness and the use of cognitive resources) (19). Cognitive trait anxiety reduces the quality of performance for musicians, especially when the attention to the technical aspect of the task has been emphasized to the detriment of the music aspect (21). Anxiety has been shown to be significantly influential for memory, with sport psychologists attributing the mechanism of impaired performance to distraction and explicit monitoring for attention (22). Distraction leads to a depletion of attentional resources because working memory gets engaged with intrusive worries, making it difficult to focus on the task at hand. Multimodal interventions, including a combination of cognitive-behavioral techniques, relaxation techniques, and breathing techniques, have shown promise in managing MPA effectively (23).

The bassoon offers specific respiratory challenges that make sustained breathing support, periodic expiration between phrases, as well as pressure control through a double reed, create a performance scenario whereby breathing patterns can impact anxiety levels as well as cognitive capacity (10). The connections between breathing patterns and cognitive function during musical performance carry implications for the development of breathing-based strategies aimed at reducing performance anxiety and maximizing cognitive function during musical performance.

The purpose of this study is to investigate the correlations between breathing patterns, performance anxiety, and cognitive skills in bassoon musicians via a cross-sectional study using a questionnaire. It also aims to investigate the degree to which performance anxiety is a mediator in the association between breathing patterns and cognitive skills, in addition to comparing the variables between professionals and non-professionals. Based on existing theoretical and empirical evidence, the following hypotheses are proposed:

H1: Dysfunctional respiratory patterns are positively associated with performance anxiety among bassoon players.

H2: Dysfunctional respiratory patterns are negatively associated with cognitive function.

H3: Performance anxiety mediates the relationship between respiratory patterns and cognitive function.

H4: Professional bassoon players demonstrate better respiratory patterns and lower anxiety levels compared to amateur players.

## 2 Materials and methods

### 2.1 Study design and participants

A cross-sectional questionnaire survey design was used to examine the relationship between breathing patterns, performance anxiety, and cognitive function among bassoon players. Participants were recruited from various sources such as online forums for music lovers, conservatoire networks, professional organizations of orchestras, and online networking sites for woodwind performers between March and May 2025. The criteria for eligible participants included those who were 18 years or older, had experience on the bassoon for at least two years, were actively engaged in the performance of the bassoon either professionally or as an amateur, and could fill out the questionnaire either in English or Chinese. Participants were excluded if they had diagnosed respiratory disorders such as asthma or chronic obstructive pulmonary disease, were currently under psychiatric care for anxiety or depression, had not performed in public within the last 12 months, or had incomplete questionnaire data.

Sample size was determined using G*Power 3.1 software based on an anticipated medium effect size (f^2^ = 0.15) for multiple regression analysis with five predictors, a statistical power of 0.80, and an alpha level of 0.05, yielding a minimum required sample of 92 participants (24). A target sample size of 120 participants was considered to allow for incomplete data and to enable sufficient power to detect subgroup differences. Data collection methods included an online survey. All the participants provided informed consent before the administration of the questionnaire, which was done by selecting the agreement button after reading the information sheet. The information sheet contained details about the purpose, procedures, voluntary nature, right to withdraw, and the steps taken to maintain the confidentiality and anonymity of the responses. The administration of the questionnaire took about 15 to 20 minutes. Participants were instructed to complete the questionnaire at a neutral time point unrelated to an imminent performance, in order to capture their general experience of breathing patterns and performance anxiety rather than acute pre-performance states. Formal ethical approval was not required for this anonymous online survey study in accordance with institutional research policies.

**Figure 1:**
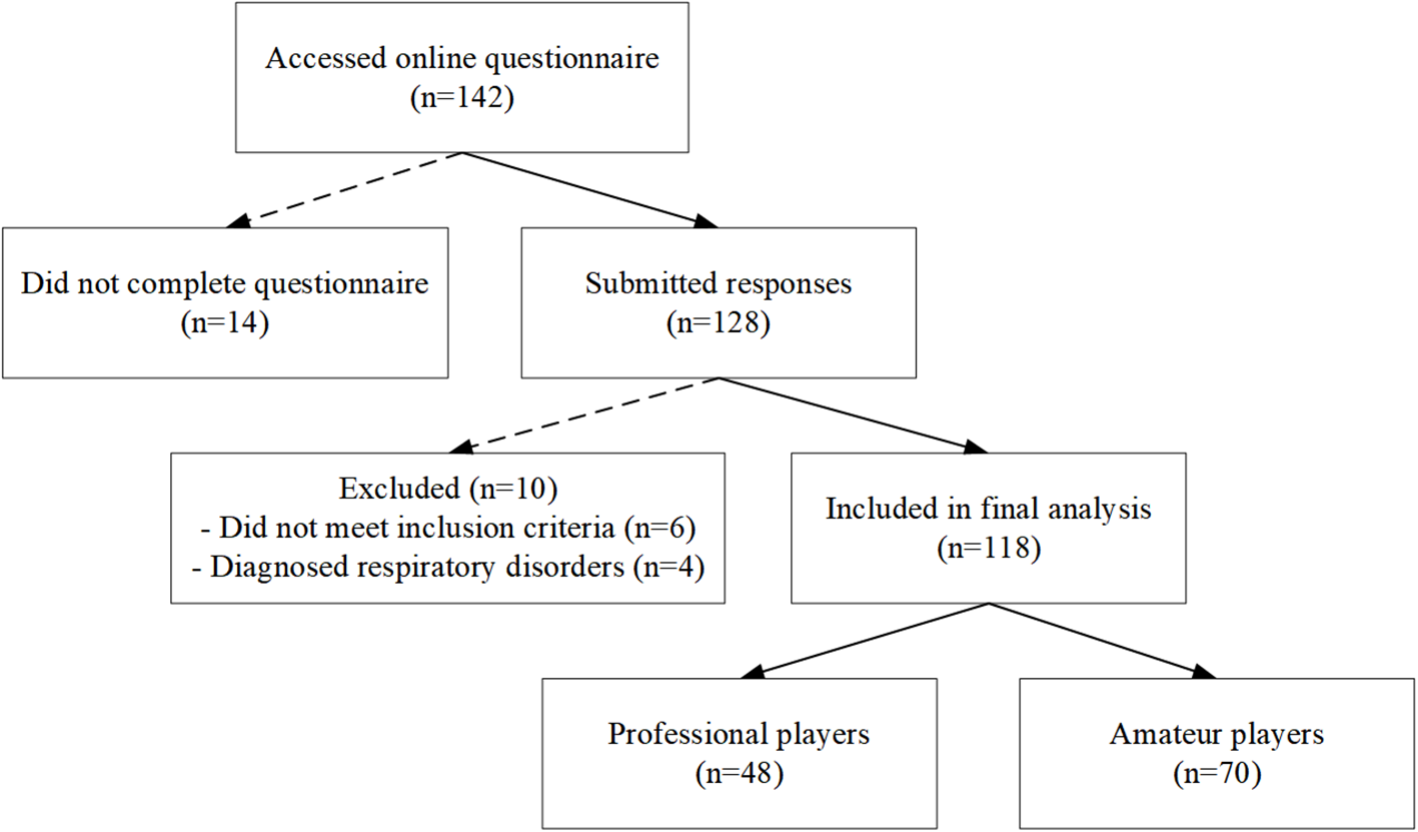
Participant screening flowchart.

A total of 142 participants gained access to the online questionnaire, of whom 128 filled the online questionnaire completely, yielding a response rate of 90.1%. After the exclusion criteria were used, 6 participants were excluded for not fulfilling the criteria for inclusion, and 4 had diagnosed cases of respiratory diseases, resulting in 118 for analysis. The participants were divided into professional and amateur groups based on their self-classification. The professional players were those who earned their main source of income from playing the bassoon or are professional players in an orchestra or ensembles (n = 48) and the amateur players were those who play the bassoon in their leisure time (n = 70). The questionnaire covered demographic variables such as age, gender, nationality, and educational attainment, as well as musical background factors such as years of experience playing the bassoon, amount of practice per week, rate of public performances over the past year, main setting for performances, and experience with breathing exercises or relaxation practices. The participant screening process is shown in **Figure 1**.

### 2.2 Measurement instruments

#### 2.2.1 Respiratory pattern assessment

Dysfunctional breathing was measured by the use of the Self-Evaluation of Breathing Questionnaire (SEBQ), which is a 25-item scale that was designed for the measurement of symptoms and the severity associated with dysfunctional breathing (25). Each item is rated on a 3-point scale (0 = never, 1 = sometimes, 2 = frequently), and the total score can range from 0 to 75, with higher scores representing higher levels of dysfunctional breathing. The SEBQ has a high level of internal consistency (Cronbach’s α = 0.93) and excellent test-retest reliability (ICC = 0.89), and captures two dimensions of breathing symptoms: sensations of air hunger and perceptions of inappropriate or restricted breathing (26).

#### 2.2.2 Performance anxiety

Performance anxiety was measured using the Kenny Music Performance Anxiety Inventory-Revised (K-MPAI-R), a 40-item self-report questionnaire grounded in Barlow’s emotion-based theory of anxiety (27). Items are rated on a 7-point Likert scale ranging from 0 (strongly disagree) to 6 (strongly agree), with total scores ranging from 0 to 240, where higher scores indicate greater levels of performance anxiety. The K-MPAI-R has been translated into 22 languages and displays high psychometric properties with diverse musicians, where the coefficients exceed 0.90 (28). The Chinese version was used on participants who preferred answering in Chinese.

#### 2.2.3 Cognitive function

Attentional control was measured using the Attentional Control Scale (ACS), which is a 20-item scale intended for the measurement of individual differences in the voluntary control of attention (29). The scale uses a 4-point Likert scale (1 = almost never to 4 = always), and the total scores range from 20 to 80, where high scores represent high attentional control ability. The ACS consists of two subscales: attentional focusing (9 items), which measures the ability to maintain attention despite distraction, and attentional shifting (11 items), which measures the ability to shift attention between tasks. The scale has been shown to have satisfactory internal consistency (α = 0.81-0.87), and has been widely used in studies of anxiety-cognition linkages (30).

#### 2.2.4 Self-rated performance

The subjective evaluation of the participants’ performance quality was determined through a single-item instrument, asking about their overall level of satisfaction with their bassoon playing over the past three months, on a 10-point rating scale (1 = extremely dissatisfied to 10 = extremely satisfied). There is support from previous literature to suggest that single-item indicators of performance satisfaction are valid for the assessment of global self-perceptions in music psychology (31). Participants indicated their perceived frequency of performance errors during recent performances using a 5-point scale (1 = never to 5 = very frequently).

### 2.3 Statistical analysis

All the analyses of statistics were performed using SPSS version 26.0, with the PROCESS macro version 4.1. The data was assessed for missing values, outliers, and normal distribution prior to any test of hypotheses. The means, standard deviations, and frequencies of all variables in the research were established. The Cronbach alpha values measured the scales’ internal consistency. Pearson correlation tests explored the bivariate correlations between all variables of respiratory style, performance anxiety, attentional control, and subjective performance. t-tests assessed differences between the groups of professionals and amateur musicians in all main variables. To examine the predictive relationships as specified in the hypotheses, a series of hierarchical regression analyses were performed. The demographic and musical background variables were entered as covariates in the first step of the regression analyses. The mediating effects of performance anxiety between respiratory patterns and cognitive functions were examined using the PROCESS macro (Model 4), with 5,000 bootstrap resamples and bias-corrected 95% confidence intervals (32). The mediation was considered to be significant if the confidence interval of the indirect effect did not contain zero. Effect size was assessed according to standard criteria, whereby f^2^ = 0.02, 0.15, and 0.35 represented small, medium, and large effects, respectively. Significance level was set to p < 0.05.

## 3 Results

### 3.1 Sample characteristics

The final sample consisted of 118 bassoonists, with 48 (40.7%) being professional and 70 (59.3%) being amateur bassoonists. The average age was 29.4 years (SD = 9.7), with a slight predominance of females (55.9%). Professional bassoonists tended to be older than amateur bassoonists (M = 34.2 vs. 26.1 years), had more experience (M = 17.8 vs. 9.0 years), practiced more hours per week (M = 19.8 vs. 10.4 hours), and performed more often within the last 12 months (M = 17.6 vs. 7.0 times). Professional bassoonists tended to perform more often in an orchestra (45.8% vs. 17.1%) and had experience with breathing or relaxation exercises (58.3% vs. 37.1%). These data are summarized in greater detail in **Table 1**.

**Table 1:** Demographic and musical background characteristics of participants (N = 118).

Variable	Total (N = 118)	Professional (n = 48)	Amateur (n = 70)
Age (years), M (SD)	29.4 (9.7)	34.2 (9.8)	26.1 (8.2)
Gender, n (%)			
Male	52 (44.1)	24 (50.0)	28 (40.0)
Female	66 (55.9)	24 (50.0)	42 (60.0)
Education, n (%)			
High school or equivalent	23 (19.5)	3 (6.3)	20 (28.6)
Bachelor’s degree	58 (49.2)	22 (45.8)	36 (51.4)
Master’s degree	31 (26.3)	19 (39.6)	12 (17.1)
Doctoral degree	6 (5.1)	4 (8.3)	2 (2.9)
Playing experience (years), M (SD)	12.6 (8.9)	17.8 (9.4)	9.0 (6.5)
Weekly practice hours, M (SD)	14.2 (9.5)	19.8 (8.7)	10.4 (8.1)
Performances in past 12 months, M (SD)	11.3 (10.2)	17.6 (11.3)	7.0 (6.4)
Primary performance context, n (%)			
Orchestral	34 (28.8)	22 (45.8)	12 (17.1)
Chamber music	29 (24.6)	14 (29.2)	15 (21.4)
Solo	18 (15.3)	8 (16.7)	10 (14.3)
Mixed	37 (31.4)	4 (8.3)	33 (47.1)
Prior breathing/relaxation training, n (%)	54 (45.8)	28 (58.3)	26 (37.1)

M, mean; SD, standard deviation.

### 3.2 Descriptive statistics of main variables

All scales demonstrated adequate to good internal consistency in the present sample, with Cronbach’s α coefficients ranging from 0.76 to 0.92. The average SEBQ score was 22.4 (SD = 12.6), which indicates a moderate level of dysfunctional breathing symptoms. There was a large range of scores; approximately 27.1% of the participants scored above a level of 30, which is a level of concern for breathing dysfunction. The range of K-MPAI-R scores was 18-198. The average was 97.3 (SD = 42.8). There was a large range of scores; approximately one-third of the participants (32.2%) scored above 120, indicating elevated levels of music performance anxiety warranting clinical attention. The average score on the ACS was 52.6 (SD = 10.3), reflecting a moderate level of attentional control capacity. The data from the focusing scale (M = 23.1, SD = 5.4) and shifting scale (M = 29.5, SD = 6.2) indicated that the participants found slightly more difficult the task of attentional focusing than shifting between tasks. Participants were moderately satisfied with their performance quality (M = 6.4, SD = 1.8) and perceived their error frequency as being relatively low (M = 2.5, SD = 0.9). Analysis of the scores’ distribution revealed the study variables were close to the normal distribution. Skewness and kurtosis were within acceptable levels of no more than ±2.0. This justified the use of parametric statistical analysis. The reliability coefficients of the variables of the study appear in **Table 2**.

**Table 2:** Descriptive statistics and internal consistency of main study variables (N = 118).

Variable	M	SD	Observed range	Possible range	Skewness	Kurtosis	Cronbach’s α
SEBQ	22.4	12.6	2–58	0–75	0.83	0.12	0.89
K-MPAI-R	97.3	42.8	18–198	0–240	0.24	-0.71	0.92
ACS Total	52.6	10.3	28–76	20–80	-0.18	-0.42	0.84
ACS Focusing	23.1	5.4	10–35	9–36	-0.31	-0.53	0.76
ACS Shifting	29.5	6.2	14–43	11–44	-0.14	-0.38	0.79
Performance Satisfaction	6.4	1.8	2–10	1–10	-0.46	-0.27	—
Performance Errors	2.5	0.9	1–5	1–5	0.58	0.34	—

M, mean; SD, standard deviation; SEBQ, Self-Evaluation of Breathing Questionnaire; K-MPAI-R, Kenny Music Performance Anxiety Inventory-Revised; ACS, Attentional Control Scale.

### 3.3 Correlations among main variables

Pearson correlation analysis was conducted to test bivariate correlations between respiratory patterns, performance anxiety, attention control, and self-perceived performance variables. Results revealed significant correlations among most study variables in the expected directions, providing preliminary support for the proposed relationships. The correlation matrix is shown in **Table 3**.

**Table 3:** Correlations among main study variables (N = 118).

Variable	1	2	3	4	5	6	7
1. SEBQ	—						
2. K-MPAI-R	0.52***	—					
3. ACS Total	-0.39***	-0.58***	—				
4. ACS Focusing	-0.34***	-0.51***	0.89***	—			
5. ACS Shifting	-0.36***	-0.53***	0.91***	0.62***	—		
6. Performance Satisfaction	-0.28**	-0.47***	0.41***	0.19*	0.38***	—	
7. Performance Errors	0.17	0.44***	-0.38***	-0.32***	-0.21*	-0.56***	—

SEBQ, Self-Evaluation of Breathing Questionnaire; K-MPAI-R, Kenny Music Performance Anxiety Inventory-Revised; ACS, Attentional Control Scale. *p < 0.05, **p < 0.01, ***p < 0.001.

Dysfunctional breathing (SEBQ) showed a strong and positive association with performance anxiety (K-MPAI-R) (r = 0.52, p < 0.001), indicating that those who exhibited higher dysfunctional breathing patterns also exhibited higher music performance anxiety, thereby testing H1. The SEBQ scores were found to have a significant negative relationship with attentional control (ACS Total) (r = -0.39, p < 0.001). This was again found in the focusing component (r = -0.34, p < 0.001) as well as the shifting component (r = -0.36, p < 0.001), offering preliminary support for H2. Performance anxiety as measured through the K-MPAI-R was strongly related to attentional control (r = -0.58, p < 0.001), as suggested by Attentional Control Theory.

Regarding the variables of performance outcome, high levels of anxiety were related to lower satisfaction with performance (r = -0.47, p < 0.001) and more frequent self-perceived errors of performance (r = 0.44, p < 0.001). Similarly, attentional control was found to be positively related to satisfaction with performance (r = 0.41, p < 0.001) and negatively related to self-perceived errors of performance (r = -0.38, p < 0.001). Dysfunctional breathing correlated negatively with satisfaction with performance (r = -0.28, p = 0.002), while the relationship with self-perceived errors of performance tended to be nonsignificant (r = 0.17, p = 0.063).

### 3.4 Predictors of performance anxiety and attentional control

Hierarchical multiple regression analyses were conducted to examine the predictive variables of performance anxiety and attentional control. For the regression analyses, the demographic variables (age, gender, years of playing experience, and hours of practice per week) were entered as covariates at Step 1, followed by the predictor variables at Step 2.

For performance anxiety (K-MPAI-R), the demographic covariates in Step 1 accounted for 11.3% of the variance (R^2^ = 0.113, F(4, 113) = 3.61, p = 0.008), with years of playing experience (β = -0.24, p = 0.018) emerging as a significant negative predictor. After adding dysfunctional breathing (SEBQ) in Step 2, the model explained an additional 23.1% of the variance (ΔR^2^ = 0.231, ΔF(1, 112) = 38.42, p < 0.001). SEBQ proved to be an important positive predictor of performance anxiety (β = 0.49, p < 0.001), meaning that the more dysfunctional breathing, the more performance anxiety, after accounting for the other variables. The model accounted for 34.4% of the variance in performance anxiety scores. These results confirm H1.

For attentional control (ACS Total), demographic covariates in Step 1 explained 8.7% of the variance (R^2^ = 0.087, F(4, 113) = 2.69, p = 0.035), with no individual predictor reaching statistical significance. In Step 2, dysfunctional breathing (SEBQ) was entered and explained an additional 12.4% of the variance (ΔR^2^ = 0.124, ΔF(1, 112) = 17.28, p < 0.001), with SEBQ negatively predicting attentional control (β = -0.36, p < 0.001). In Step 3, performance anxiety (K-MPAI-R) was added to examine whether anxiety contributed to attentional control beyond breathing patterns. This step explained an additional 18.6% of the variance (ΔR^2^ = 0.186, ΔF(1, 111) = 32.15, p < 0.001). In the final model, performance anxiety emerged as the strongest predictor of attentional control (β = -0.48, p < 0.001), while the effect of SEBQ was reduced but remained marginally significant (β = -0.17, p = 0.046). The explained variance for the final model was 39.7% of the total variance in attentional control, which supports H2, suggesting the mediating role of performance anxiety. The results of the regression analysis are presented in **Table 4**.

**Table 4:** Hierarchical regression analyses predicting performance anxiety and attentional control.

Predictor	Performance anxiety (K-MPAI-R)			Attentional control (ACS Total)		
	β	SE	p	β	SE	p
Step 1						
Age	-0.08	0.42	0.461	0.11	0.10	0.318
Gender	0.12	7.84	0.178	-0.07	1.92	0.452
Playing experience	-0.24	0.47	0.018	0.14	0.12	0.187
Weekly practice hours	-0.09	0.43	0.352	0.08	0.11	0.406
R^2^	0.113		0.008	0.087		0.035
Step 2						
SEBQ	0.49	0.28	<0.001	-0.36	0.07	<0.001
ΔR^2^	0.231		<0.001	0.124		<0.001
Step 3						
K-MPAI-R	—	—	—	-0.48	0.02	<0.001
ΔR^2^	—		—	0.186		<0.001
Final Model						
Total R^2^	0.344			0.397		

SEBQ, Self-Evaluation of Breathing Questionnaire; K-MPAI-R, Kenny Music Performance Anxiety Inventory-Revised; ACS, Attentional Control Scale. Gender coded as 0 = male, 1 = female.

### 3.5 Mediating effect of performance anxiety

To test H3, a mediation analysis was conducted to examine whether performance anxiety mediates the association between dysfunctional breathing and attentional control. Results indicated that dysfunctional breathing (SEBQ) was significantly associated with the mediator, performance anxiety (K-MPAI-R) (a path: B = 1.76, SE = 0.28, p < 0.001). Performance anxiety was significantly associated with attentional control (ACS) after controlling for dysfunctional breathing (b path: B = -0.11, SE = 0.02, p < 0.001). The total effect of dysfunctional breathing on attentional control was significant (c path: B = -0.32, SE = 0.07, p < 0.001), indicating that higher levels of dysfunctional breathing were associated with lower attentional control. When performance anxiety was included in the model, the direct effect of dysfunctional breathing on attentional control was reduced and became marginally significant (c’ path: B = -0.13, SE = 0.07, p = 0.048).

**Figure 2:**
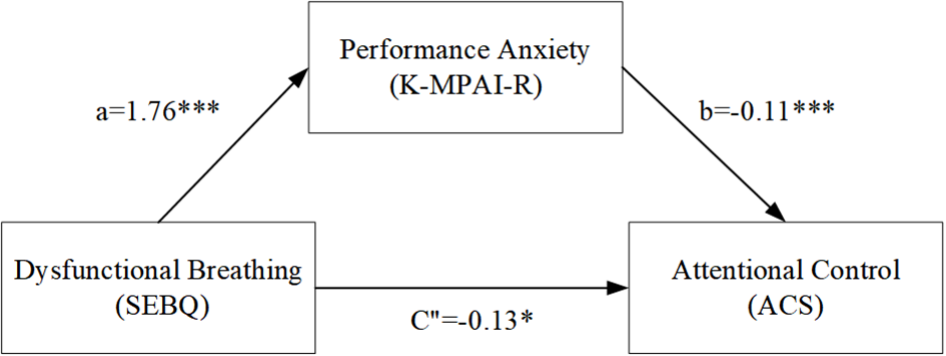
The mediating role of performance anxiety. *p < 0.05, ***p < 0.001.

The indirect effect of dysfunctional breathing on attentional control through performance anxiety was significant (ab = -0.19, SE = 0.05, 95% CI [-0.30, -0.10]), as the confidence interval did not include zero. The ratio of the indirect effect to the total effect indicated that performance anxiety accounted for approximately 59.4% of the total effect of dysfunctional breathing on attentional control. These findings provide support for H3, suggesting that performance anxiety partially mediates the relationship between dysfunctional breathing patterns and cognitive function in bassoonists. The mediation model is shown in **Figure 2**.

### 3.6 Subgroup comparisons

Independent samples t-tests were conducted to compare professional and amateur bassoon players on the main study variables to test H4. Professional players reported significantly lower levels of dysfunctional breathing (M = 18.2, SD = 10.8) compared to amateur players (M = 25.3, SD = 13.1), t(116) = -3.07, p = 0.003, Cohen’s d = 0.58, indicating that professional musicians demonstrate better respiratory patterns. Professional players also demonstrated significantly higher attentional control (M = 55.8, SD = 9.6) than amateur players (M = 50.4, SD = 10.3), t(116) = 2.82, p = 0.006, Cohen’s d = 0.54. This pattern was consistent across both subscales, with professional players scoring higher on attentional focusing (M = 24.6, SD = 5.1 vs. M = 22.1, SD = 5.5), t(116) = 2.48, p = 0.015, Cohen’s d = 0.47, and attentional shifting (M = 31.2, SD = 5.8 vs. M = 28.3, SD = 6.3), t(116) = 2.52, p = 0.013, Cohen’s d = 0.48. Regarding performance outcomes, professional players reported higher performance satisfaction (M = 7.1, SD = 1.6) than amateur players (M = 5.9, SD = 1.8), t(116) = 3.64, p < 0.001, Cohen’s d = 0.71, and fewer performance errors (M = 2.2, SD = 0.8) compared to amateurs (M = 2.7, SD = 0.9), t(116) = -3.01, p = 0.003, Cohen’s d = 0.59.

**Figure 3:**
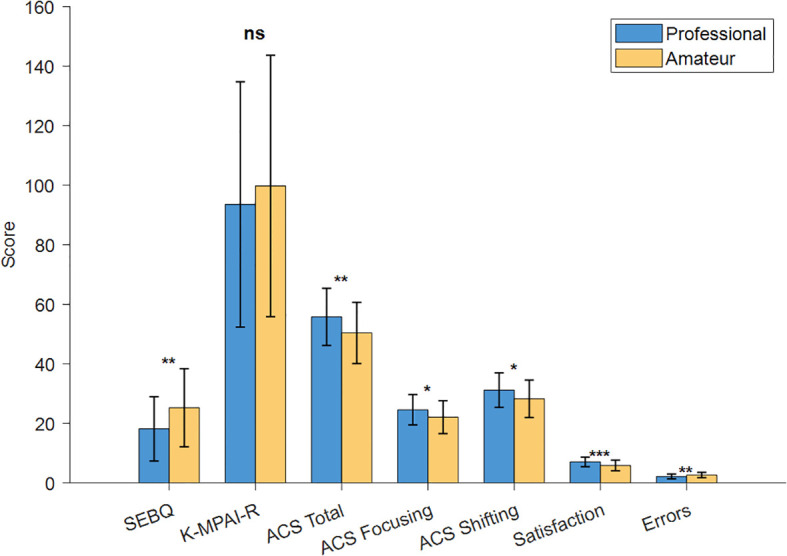
Comparison of main study variables between professional and amateur players. Error bars represent standard deviations. ns, not significant; *p < 0.05, **p < 0.01, ***p < 0.001.

Contrary to expectations, performance anxiety did not differ significantly between professional (M = 93.6, SD = 41.2) and amateur players (M = 99.8, SD = 43.9), t(116) = -0.76, p = 0.448, Cohen’s d = 0.15. The results partially support H4. Professional bassoon players demonstrated better respiratory control and attentional ability compared to amateur bassoonists; however, anxiety levels were comparable between the two groups. The group comparison is represented in **Figure 3**.

## 4 Discussion

The current investigation has explored the relationship between respiration patterns, performance anxiety, and cognition in bassoon players, adding original knowledge with respect to the psychophysiological process involved in the performance of wind instruments. The findings presented in the article support the theoretical approach proposed.

There is a strong positive association between dysfunctional breathing and performance anxiety (r = 0.52), confirming H1 and consistent with previous findings about the mutual relationship existing between breathing patterns and emotional experiences (17). It seems particularly evident among bassoon musicians, as the playing of this instrument necessitates the constant support of breathing effort and the regulation of air pressure through the double reed and the intermittent expulsion of air between musical phrases. The strength of this correlation is stronger than what has been generally reported regarding general musician groups, suggesting that the respiratory-anxiety association may be amplified when breathing serves both physiological and technical performance functions simultaneously (12). Previous research showed that music students with high levels of anxiety tend to sigh more and have breathing irregularities before and during performances, which corresponds to the dysfunctional breathing patterns found among the sample (33).

The negative correlation between dysfunctional breathing and attentional control (r = -0.39) supports H2 and expands the current knowledge about the association between dysfunctional respiration and cognition. Neuroimaging results show that dysfunctional breathing has a direct impact on the functional connectivity of the brain and affects cognition, including attention, memory, and decision-making, through changes in cortical excitability (34). What the present findings suggest is that those bassoonists presenting dysfunctional breathing symptoms may be allocating fewer attentional resources to the complex cognitive demands of music performance. This is consistent with previous findings, which suggest that exercises focusing on diaphragmatic breathing can lead to improvements not only in breathing function but also cognitive performance among patients requiring better attention (35).

The mediation analysis showed that performance anxiety accounted for 59.4% of the association between dysfunctional breathing and attentional control, confirming H3 and representing the most theoretically significant finding of this study. The proposed mediation pathway is consistent with Attentional Control Theory, which proposes that anxiety impairs the effective functioning of the goal-driven attentional system by increasing vulnerability to stimulus-driven attention to threat-related stimuli (19, 20). The presence of partial mediation in the findings shows that there are two pathways through which dysfunctional breathing is associated with cognitive function: first through the indirect route of increased anxiety and then through the direct route, potentially involving biological pathways such as reduced blood oxygenation to the brain or imbalances in the autonomic nervous system. Previous research highlights the impact of breathing on rhythmic brain activity and the modulation of the power of higher frequency waves that reflect cognitive activity (36). The practice of slow and regulated breathing has been found to increase the activity of the parasympathetic nervous system and heart rate variability, which are physiological responses that work to balance the sympathetic activation that occurs in anxiety (13).

The comparison of professional and amateur bassoon players partially supports H4. The professional players demonstrated significantly more optimal breathing, control of attention, and performance achievement, with medium effect sizes (d = 0.47-0.71) indicating practically meaningful differences. These findings suggest that extensive experience in training and performing is associated with more optimal breathing patterns and stronger attentional control. The trial with woodwind conservatory students proved that the integration of breathing muscle training with nutritional treatment significantly improved both respiratory function and emotional regulation, supporting the trainability of breathing patterns in wind musicians (37).

The fact that no significant differences exist in performance anxiety between professionals and amateurs challenges the assumption that experience serves as a buffer against performance-related anxiety. Rather, these findings suggest that professional expertise may not eliminate anxiety but instead enhance musicians’ capacity to regulate or function effectively under comparable levels of activation. Consistent with the effectiveness-efficiency distinction in Attentional Control Theory (19), professional bassoonists may maintain superior performance outcomes at a comparable level of anxiety, potentially through more refined regulatory strategies and greater attentional control. This finding is in line with the findings obtained by Spahn et al., suggesting that professionals exhibit similar or even greater levels of anxiety compared to amateurs, which could be linked to the performance pressures inherent in the professionals’ occupation (38). The same group of researchers also found that professional orchestra players were more likely to be grouped under the anxiety profiles characterized by chronic symptoms and impaired functional coping compared to amateur players (5). A similar interpretation regarding the role of investment and the musical self-concept related to professional status and performance anxiety was also proposed (39).

The results have important implications for music pedagogy and performance psychology. The strong relationship between dysfunctional breathing and both anxiety and cognitive impairment suggests that the use of respiratory patterns as an intervention strategy for performance anxiety in wind musicians is worth considering. A meta-analysis has shown that there is significant stress and anxiety symptom relief from breathwork interventions, and that this is mediated by autonomic nervous system function and heart rate variability (40). Previous research found that breathing exercises with slow breathing rates led to a reduction in state anxiety and improvements in physiological regulation for highly anxious musicians in a single training session, which suggests that relatively short procedures can be effective (41). Another meta-analysis identified breathing techniques among the most commonly employed approaches for managing music performance anxiety, alongside cognitive restructuring and physical relaxation methods (23).

The current research theoretically combines Attentional Control Theory with respiratory physiology to explain possible directions for the development of multimodal interventions. The mediation pathway identified in the current research suggests that interventions targeting dysfunctional breathing may be associated with reductions in performance anxiety and, in turn, improvements in attentional control, likely resulting in enhanced performance quality. A systematic review supports the fact that multimodal interventions with cognitive-behavioral, relaxation, and breathing techniques have been found to be effective in managing music performance anxiety (42). Given the unique respiratory demands of bassoon performance, instrument-specific breathing training protocols may warrant development and empirical evaluation.

However, the proposed mediation model should be interpreted with caution, as the cross-sectional design does not allow causal inference regarding the directionality of the relationship between the variables. The mediation pathway is hypothetical, and the bidirectional relationship between breathing and emotions suggests that chronic anxiety may also contribute to the development of dysfunctional breathing (17). All primary constructs were assessed via self-report, which introduces the possibility of common method variance and may have inflated the observed correlations between variables. Objective measures of breathing function during actual performance situations would help to increase construct validity. The timing of questionnaire completion relative to performance events was not controlled beyond instructing participants to respond at a neutral time point, which may have introduced variability in responses. While the sample size was sufficient for the analyses that were carried out, it does represent only a small proportion of the total number of people in the world who play the bassoon, and the recruitment through online communities may have introduced selection biases.

Future studies should examine these limitations with prospective designs that include physiological data regarding breathing pattern during performance. There would also be practical value to intervention studies regarding the efficacy of breathing pattern training for bassoon players. Comparative studies involving various wind instruments could help determine the generality of the findings regarding the relationship between the unique breathing requirements of the bassoon and the results to wind musicians more generally. There would also be value to further investigation regarding the unexpected finding regarding the level of anxiety between professional and amateur players, potentially through qualitative methods exploring the subjective experience and meaning of performance anxiety across career stages.

## 5 Conclusion

This study explored the relationship between breathing patterns, performance anxiety, and cognitive functioning in 118 bassoon players. The results showed that dysfunctional breathing had a positive relationship with performance anxiety and a negative relationship with attentional control. In addition, the study found that performance anxiety partially mediated the relationship between dysfunctional breathing and attentional control, explaining 59.4% of the total effect. The findings of this study provide support for the use of Attentional Control Theory in the context of wind instrument playing. The professionals demonstrated more beneficial respiratory patterns, higher attentional control, and better performance achievement compared to nonprofessionals. There were no significant differences regarding performance anxiety. This suggests that being a professional does not provide any protection against performance-related anxiety. These results highlight the importance of respiratory patterns to explain the relationship between anxiety and cognition in wind players, as well as that interventions related to breathing could be a promising approach to address performance anxiety and improve cognition.

## Data Availability

The original contributions presented in the study are included in the article/supplementary material. Further inquiries can be directed to the corresponding author.

## References

[1] Fernholz I Mumm JL Plag J Noeres K Rotter G Willich SN . Performance anxiety in professional musicians: a systematic review on prevalence, risk factors and clinical treatment effects. Psychol Med. (2019) 49:2287–306. doi: 10.1017/s0033291719001910 31474244

[2] Thomas JP Nettelbeck T . Performance anxiety in adolescent musicians. Psychol Music. (2014) 42:624–34. doi: 10.1177/0305735613485151

[3] Gómez-López B Sánchez-Cabrero R . Current trends in music performance anxiety intervention. Behav Sci. (2023) 13:720. doi: 10.3390/bs13090720 37753998 PMC10525579

[4] Kenny D . The psychology of music performance anxiety. Oxford, UK: Oxford University Press (2011).

[5] Spahn C Tenbaum P Immerz A Hohagen J Nusseck M . Dispositional and performance-specific music performance anxiety in young amateur musicians. Front Psychol. (2023) 14:1208311. doi: 10.3389/fpsyg.2023.1208311 37583605 PMC10425269

[6] Herman R Clark T . It’s not a virus! reconceptualizing and de-pathologizing music performance anxiety. Front Psychol. (2023) 14:1194873. doi: 10.3389/fpsyg.2023.1194873 38022988 PMC10667921

[7] Becher L Gena AW Alsaad H Richter B Spahn C Voelker C . The spread of breathing air from wind instruments and singers using schlieren techniques. Indoor Air. (2021) 31:1798–814. doi: 10.1111/ina.12869 34121229

[8] Fletcher N . The physiological demands of wind instrument performance. Acoust Aust. (2000) 28:53–6.

[9] Zuskin E Mustajbegovic J Schachter EN Kern J Vitale K Pucarin-Cvetkovic J . Respiratory function in wind instrument players. Med del Lavoro. (2009) 100:133. 19382523

[10] Spahn C Hipp AM Schubert B Axt MR Stratmann M Schmölder C . Airflow and air velocity measurements while playing wind instruments, with respect to risk assessment of a SARS-CoV-2 infection. Int J Environ Res Public Health. (2021) 18:5413. doi: 10.3390/ijerph18105413 34069419 PMC8159134

[11] Chang X . (2018). “ Analysis of embouchure and breath control of bassoon”, in: 2018 International Conference on Culture, Literature, Arts & Humanities (ICCLAH 2018). UK: Francis Academic Press 348–50. doi: 10.25236/icclah.18.077

[12] Sokoli E Hildebrandt H Gomez P . Classical music students’ pre-performance anxiety, catastrophizing, and bodily complaints vary by age, gender, and instrument and predict self-rated performance quality. Front Psychol. (2022) 13:905680. doi: 10.3389/fpsyg.2022.905680 35814093 PMC9263585

[13] Balban MY Neri E Kogon MM Weed L Nouriani B Jo B . Brief structured respiration practices enhance mood and reduce physiological arousal. Cell Rep Med. (2023) 4(1):100895. doi: 10.1016/j.xcrm.2022.100895 36630953 PMC9873947

[14] Chin P Gorman F Beck F Russell BR Stephan KE Harrison OK . A systematic review of brief respiratory, embodiment, cognitive, and mindfulness interventions to reduce state anxiety. Front Psychol. (2024) 15:1412928. doi: 10.3389/fpsyg.2024.1412928 38933581 PMC11203600

[15] Chaitanya S Datta A Bhandari B Sharma VK . Effect of resonance breathing on heart rate variability and cognitive functions in young adults: a randomised controlled study. Cureus. (2022) 14(2):e22187. doi: 10.7759/cureus.22187 35308668 PMC8924557

[16] Little AL . The A52 breath method: a narrative review of breathwork for mental health and stress resilience. Stress Health. (2025) 41:e70098. doi: 10.1002/smi.70098 40792649 PMC12341363

[17] Grassmann M Vlemincx E Von Leupoldt A Mittelstädt JM Van den Bergh O . Respiratory changes in response to cognitive load: a systematic review. Neural Plast. (2016) 2016:8146809. doi: 10.1155/2016/8146809 27403347 PMC4923594

[18] Maleki A Ravanbakhsh M Saadat M Bargard MS Latifi SM . Effect of breathing exercises on respiratory indices and anxiety level in individuals with generalized anxiety disorder: a randomized double-blind clinical trial. J Phys Ther Sci. (2022) 34:247–51. doi: 10.1589/jpts.34.247 35400836 PMC8989478

[19] Eysenck MW Derakshan N . New perspectives in attentional control theory. Pers Individ Dif. (2011) 50:955–60. doi: 10.1016/j.paid.2010.08.019 38826717

[20] Eysenck MW Derakshan N Santos R Calvo MG . Anxiety and cognitive performance: attentional control theory. Emotion. (2007) 7:336. doi: 10.1037/1528-3542.7.2.336 17516812

[21] Hörster A Hansen J . Self-consciousness and trait anxiety influence music performance in high-pressure situations. Musicae Scientiae. (2024) 28:758–78. doi: 10.1177/10298649241249667

[22] Matei R Ginsborg J . Music performance anxiety in classical musicians–what we know about what works. BJPsych Int. (2017) 14:33–5. doi: 10.1192/s2056474000001744 29093935 PMC5618811

[23] Faur AL Pintea S Vaida S Opre AN . The efficacy of cognitive and behavioral interventions upon music performance anxiety: a meta-analysis. Psychol Music. (2023) 51:357–72. doi: 10.1177/03057356221115461

[24] Faul F Erdfelder E Buchner A Lang A-G . Statistical power analyses using G* Power 3.1: tests for correlation and regression analyses. Behav Res Methods. (2009) 41:1149–60. doi: 10.1201/9781351204750-8 19897823

[25] Courtney R Greenwood KM . Preliminary investigation of a measure of dysfunctional breathing symptoms: the self evaluation of breathing questionnaire (SEBQ). Int J Osteopathic Med. (2009) 12:121–7. doi: 10.1016/j.ijosm.2009.02.001 38826717

[26] Mitchell A Bacon C Moran R . Reliability and determinants of self-evaluation of breathing questionnaire (SEBQ) score: a symptoms-based measure of dysfunctional breathing. Appl Psychophysiol Biofeedback. (2016) 41:111–20. doi: 10.1007/s10484-015-9316-7 26400252

[27] Kenny DT . (2009). “ The factor structure of the revised Kenny music performance anxiety inventory”, in: International Symposium on Performance Science. Utrecht, Netherlands: AEC. 37–41.

[28] Kenny DT . The Kenny music performance anxiety inventory (K-MPAI): scale construction, cross-cultural validation, theoretical underpinnings, and diagnostic and therapeutic utility. Front Psychol. (2023) 14:1143359. doi: 10.3389/fpsyg.2023.1143359 37325731 PMC10262052

[29] Derryberry D Reed MA . Anxiety-related attentional biases and their regulation by attentional control. J Abnormal Psychol. (2002) 111:225. doi: 10.1037/0021-843x.111.2.225 12003445

[30] Judah MR Grant DM Mills AC Lechner WV . Factor structure and validation of the attentional control scale. Cogn Emotion. (2014) 28:433–51. doi: 10.1080/02699931.2013.835254 24044520

[31] Papageorgi I Creech A Welch G . Perceived performance anxiety in advanced musicians specializing in different musical genres. Psychol Music. (2013) 41:18–41. doi: 10.1177/0305735611408995

[32] Hayes AF . Introduction to mediation, moderation, and conditional process analysis: a regression-based approach. New York, NY: Guilford Press (2022).

[33] Guyon AJ Cannavò R Studer RK Hildebrandt H Danuser B Vlemincx E . Respiratory variability, sighing, anxiety, and breathing symptoms in low-and high-anxious music students before and after performing. Front Psychol. (2020) 11:303. doi: 10.3389/fpsyg.2020.00303 32174869 PMC7054282

[34] Duan Y Guo X Ren B Liu F Li Y Liu F . An alternating breathing pattern significantly affects the brain functional connectivity and mood states. Front Hum Neurosci. (2025) 19:1539222. doi: 10.3389/fnhum.2025.1539222 40309665 PMC12040909

[35] Abdullahi A Wong TW Ng SS . Efficacy of diaphragmatic breathing exercise on respiratory, cognitive, and motor function outcomes in patients with stroke: a systematic review and meta-analysis. Front Neurol. (2024) 14:1233408. doi: 10.3389/fneur.2023.1233408 38283673 PMC10811179

[36] Heck DH Correia BL Fox MB Liu Y Allen M Varga S . Recent insights into respiratory modulation of brain activity offer new perspectives on cognition and emotion. Biol Psychol. (2022) 170:108316. doi: 10.1016/j.biopsycho.2022.108316 35292337 PMC10155500

[37] Sanchis C Plaza M Checa I Monleón C . Combined effects of a Mediterranean diet and respiratory muscle training on higher education woodwind musicians: a randomized controlled trial. Heliyon. (2024) 10(15):e35495. doi: 10.1016/j.heliyon.2024.e35495 39170324 PMC11336701

[38] Spahn C Krampe F Nusseck M . Classifying different types of music performance anxiety. Front Psychol. (2021) 12:538535. doi: 10.3389/fpsyg.2021.538535 33967870 PMC8102674

[39] Castiglione C Rampullo A Cardullo S . Self representations and music performance anxiety: a study with professional and amateur musicians. Europe's J Psychol. (2018) 14:792. doi: 10.5964/ejop.v14i4.1554 30555586 PMC6266529

[40] Fincham GW Strauss C Montero-Marin J Cavanagh K . Effect of breathwork on stress and mental health: a meta-analysis of randomised-controlled trials. Sci Rep. (2023) 13:432. doi: 10.1038/s41598-022-27247-y 36624160 PMC9828383

[41] Wells R Outhred T Heathers JA Quintana DS Kemp AH . Matter over mind: a randomised-controlled trial of single-session biofeedback training on performance anxiety and heart rate variability in musicians. PloS One. (2012) 7:e46597. doi: 10.1371/journal.pone.0046597 23056361 PMC3464298

[42] Bakhtiari P Nikanmajd N Ghasemi Shayan R . Recent developments in coping strategies focusing on music performance anxiety: a systematic review. Front Psychol. (2025) 16:1507229. doi: 10.3389/fpsyg.2025.1507229 40351598 PMC12062091

